# A Novel Anti-CEACAM5 Monoclonal Antibody, CC4, Suppresses Colorectal Tumor Growth and Enhances NK Cells-Mediated Tumor Immunity

**DOI:** 10.1371/journal.pone.0021146

**Published:** 2011-06-22

**Authors:** Chaogu Zheng, Jing Feng, Di Lu, Ping Wang, Shu Xing, Jean-Luc Coll, Dongling Yang, Xiyun Yan

**Affiliations:** 1 National Laboratory of Biomacromolecules, Institute of Biophysics, Chinese Academy of Sciences, Beijing, People's Republic of China; 2 Groupe de Recherche sur le Cancer du Poumon, Equipe INSERM 9924, Institut Albert Bonniot, Grenoble, France; Centre de Recherche Public de la Santé (CRP-Santé), Luxembourg

## Abstract

Carcinoembryonic antigen (CEA, CEACAM5, and CD66e) has been found to be associated with various types of cancers, particularly colorectal carcinoma, and developed to be a molecular target for cancer diagnosis and therapy. In present study, we generated a novel anti-CEACAM5 monoclonal antibody, namely mAb CC4, by immunizing mice with living colorectal cancer LS174T cells. Immunohistochemical studies found that mAb CC4 specifically and strongly binds to tumor tissues, especially colorectal adenocarcinoma. In xenografted mice, mAb CC4 is specifically accumulated in tumor site and remarkably represses colorectal tumor growth. *In vitro* functional analysis showed that mAb CC4 significantly suppresses cell proliferation, migration and aggregation of colorectal cancer cells and also raises strong ADCC reaction. More interestingly, mAb CC4 is able to enhance NK cytotoxicity against MHC-I-deficient colorectal cancer cells by blocking intercellular interaction between epithelial CEACAM5 and NK inhibitory receptor CEACAM1. These data suggest that mAb CC4 has the potential to be developed as a novel tumor-targeting carrier and cancer therapeutic.

## Introduction

After the discovery of Carcinoembryonic antigen (CEA, CEACAM5, and CD66e) as a tumor-associated antigen in human colon cancer [1], CEACAM5 has been found over-expressed in a high percentage of human tumors, including 90% of gastrointestinal, colorectal and pancreatic cancers, 70% of non-small cell lung cancer cells and 50% of breast cancers [2]. High levels of CEACAM5 have also been implicated with enhanced metastasis and the development of malignancy [3], [4]. Owing to its ectopic expression and correlation with metastatic potential in cancers, particularly colorectal cancer, CEACAM5 measurement has been widely applied in clinical detection of liver metastasis from colorectal cancers and post-surgical surveillance of colon cancer [5], [6].

Belonging to the CEACAM (CEA-related Cell Adhesion Molecule) family, a prominent group in the immunoglobulin superfamily of cell adhesion molecules (IgCAMs), CEACAM5 mainly serves as a cell adhesion molecule mediating intercellular contact by both homophilic (CEACAM5 to CEACAM5) binding and heterophilic binding (CEACAM5 to CEACAM1 or CEACAM6). These interactions are predominantly mediated by the N-terminal IgV-like domain [7], which is conserved among all the CEACAM family members. Besides its functions in cell adhesion and migration, CEACAM5 also inhibits anoikis [8], apoptosis in the absence of adhesive interactions with extracellular matrix (ECM). Since resistance to anoikis is a characteristic of tumor cells, inhibition of anoikis by CEACAM5 suggests its role in facilitating tumorigenesis and metastasis. Indeed, the tumorigenic functions of CEACAM5 had been demonstrated in both 3D culture of colon carcinoma cell lines *in vitro* [9] and CEABAC transgenic mice *in vivo* [10], [11]; and a number of studies evidenced the contribution of CEACAM5 to tumor invasion and metastasis [3], [4].

Targeting the tumor-associated expression and oncogenic functions of CEACAM5, a pile of monoclonal antibodies against CEACAM5 have been developed in previous years to facilitate the diagnosis and therapy of human cancers. However, most of these therapeutic antibodies were conjugated with radioisotype, immunotoxin, cytokine or cytotoxic enzyme [12], [13], [14], [15], which lead to considerable side effects. The availability of natural anti-CEACAM5 mAbs with significant tumor-targeting and -suppressing activity is still limited. In this paper, we report a novel anti-CEACAM5 mAb, namely CC4, which specifically accumulates in tumor tissues and remarkably inhibits tumor growth of colorectal cancer both *in vivo* and *in vitro*.

CEACAM1, one of the binding partners of CEACAM5 in the CEACAM family, has been implicated in contact-dependent regulation of immune response associated with cancer [16]. CEACAM1 contains two ITIM sequences located within its cytoplasmic tail and was found expressed on CD16-negative NK cells as a class I MHC-independent inhibitory receptor [17]. Homophilic CEACAM1 interactions between a MHC-I-deficient melanoma cell line (1106mel) and NK cells significantly inhibited NK killings against cancer cells [17]. Moreover, forced expression of CEACAM5 in HLA class I-defective 721.221 cells also suppressed NK cytotoxicity [18], indicating CEACAM5-CEACAM1 heterophilic interaction is also able to trigger inhibitory CEACAM1 signaling in NK cells. However, the function of CEACAM5 in inhibiting the killings by CD16-negative NK cells has never been tested in colorectal cancer, in which CEACAM5 displays the highest pathological expression. In present study, we show that the same tumor immune escape mechanism also exists in human colorectal cancer and mAb CC4 can significantly block the inhibition of NK cytotoxicity by interfering with the CEACAM5-CEACAM1 intercellular interaction.

## Results

### Generation and characterization of mAb CC4

**Figure 1:**
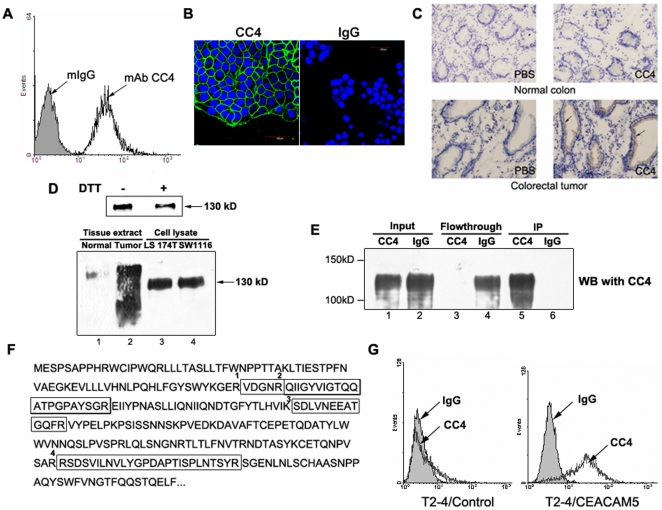
Characterization of mAb CC4 and identification of mAb CC4 antigen. MAb CC4 was used to stain LS174T cells in flow cytometry assay (A) and immunofluorescence (B). Isotype-matched murine normal IgG served as negative control. Frozen sections of normal colon and colorectal tumor tissues were stained by mAb CC4 in immunohistochemical analysis (C); and extracts from these tissues and whole cell extracts of LS174T and SW1116 were subjected to immunoblot using mAb CC4 (D, lower panels). LS174T cell lysate were immunoblotted with CC4 under reduced or non-reduced conditions (D, upper panels). Immunoprecipitation was performed using mAb CC4 or mIgG to accumulate the antigen protein and immunoprecipitates were subjected to Western blot assays (E). Four peptides were obtained from LC-MS analysis using CC4 precipitates and the antigen was identified as CEACAM5, of which partial amino acid sequence and these identified peptides were illustrated (F). Bladder cancer cells T2-4 were transfected with CEACAM5 expression plasmids and subjected to flow cytometry analysis with CC4 (G).

Monoclonal antibody CC4 was generated by immunizing mice with living human colorectal cancer cells, LS174T, and it showed strong binding to fixed LS174T cells in primary cell-ELISA screens. Several experiments were then performed to characterize mAb CC4 at the biochemical, cellular and histological levels. First of all, the binding of mAb CC4 to living LS174T cells was confirmed by flow cytometry analysis (Figure 1A), and immunofluorescence showed mAb CC4 recognizes a membrane-bound protein on cell surfaces (Figure 1B). Immunohistochemical analysis showed the specific recognition of mAb CC4 to colorectal tumor, particularly epithelial cells of rectal gland, but not the normal colon tissues from the same patient (Figure 1C). Western blot assays with tissue extracts confirmed the remarkably stronger binding of mAb CC4 to tumor than normal tissues (Figure 1D, lower panel). Cell lysates from LS174T and SW1116, another colorectal cancer cell line, were also recognized by mAb CC4 in immunoblot assays (lane 3 and 4). Furthermore, mAb CC4 recognizes the antigen under both reducing and non-reducing conditions, suggesting a linear epitope (Figure 1D, upper panel).

### Tumor specificity of mAb CC4

Bindings of mAb CC4 to various types of cancer cells were studied by flow cytometry and immunofluorescence (Table S1), and we found that all of the nine tested colorectal cancer cell lines were positive for CC4 staining. We then screened both normal and tumor tissues from several major body organs by immunohistochemistry. MAb CC4 did not stain or only slightly stained normal tissues with a frequency of staining of 31.71% (13/41, Table S2) compared to a remarkably strong recognition in 71.88% (69/96, Table S2) of various tumor tissues. Specially, all the sections from digestive system cancers tested, including colorectal carcinoma, gastric carcinoma and esophageal cancer were positive for mAb CC4 staining, whereas just 22% normal colon tissues were observably stained. Thus, these data indicated the specificity of mAb CC4 to digestive system tumors, particularly colorectal tumors at both cellular and histological levels.

### Antigen identification of mAb CC4

To identify the antigen recognized by mAb CC4, whole cell lysates from LS174T cells was immunoprecipitated by mAb CC4, and the captured antigen was then tested in Western blot assay (Figure 1E) and subjected to proteomic analysis. After trypsin digestions, four tryptic peptides (1-VDGNRQIIGYVIGTQQATPGPAYSGR, 2-QIIGYVIGTQQATPGPAYSGR, 3-SDLVNEEATGQFR, and 4-RSDSVILNVLYGPDAPTISPLNTSYR) were identified by mass spectrometry (Figure 1F). Database searching revealed that the only protein containing these sequences is carcinoembryonic antigen-related cell adhesion molecule 5 (CEACAM5, CEA or CD66e). The binding of mAb CC4 to bladder cancer T2-4 cells transfected with CEACAM5-expressing vectors further validated the recognition of mAb CC4 to CEACAM5 (Figure 1G).

### Specific targeting of mAb CC4 to tumor in vivo

**Figure 2:**
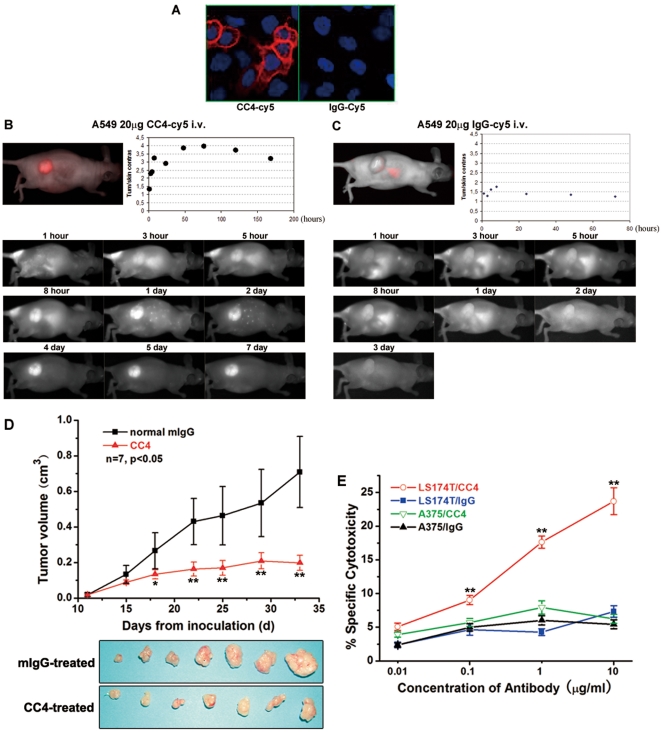
mAb CC4 was able to target xenografted tumors, suppress tumor growth in vivo, and raised ADCC reactions in vitro. Non-small cell lung cancer cells A549 was stained with cy5 labeled CC4 in immunofluorescence (A). Cells were injected to nude mice to form tumors and CC4-cy5 or IgG-cy5 was administrated intravenously. In vivo optical imaging of fluorescent antibodies was performed in the indicated times (B and C). LS174T cells were injected subcutaneously into nude mice. After forming tumor, mice were treated with CC4 or normal IgG control for indicated times. Tumors were taken out for measuring the size and calculating the volume, as well as photography (D, n = 7 in each group, **P<0.01 and *P<0.05). LS174T cells were subjected to ADCC assays using murine spleen cells as effector cells. Results for specific cytotoxicity of cancer cells were presented in bar graphs (E).

After identifying the antigen of this cancer-specific monoclonal antibody, we then employed an *in vivo* model to test whether mAb CC4 could specifically target the tumor in xenografted mice. A widely used non-small cell lung cancer cell line A549 was engaged in this study and confirmed to be positive for mAb CC4 staining in immunofluorescence assays (Figure 2A). 2×10^7^ A549 cells were injected to form a 6-8 mm-diameter tumor in nude mouse. Tumor-bearing mice then received intravenous injection of Cy5-labeled CC4 antibody or Cy5-conjugated IgG and monitored by fluorescence reflectance imaging system. As shown in Figure 2B, mAb CC4 was amazingly accumulated in the tumor site within just 8 hours after the injection. The tumor/skin contrast reached as high as 4 in 2 days and the high contrast lasted more than a week. However, the negative control, Cy5-IgG, was only found to undergo protein metabolism in liver and these control mice maintained a low tumor/skin contrast as the background (Figure 2C). These optical imaging data in an *in vivo* tumor-bearing mice model exhibit a very exciting capability of mAb CC4 in specifically targeting tumors and raise the possibility of applying mAb CC4 in clinical diagnosis.

### Inhibition of human tumor growth in xenografted mice by mAb CC4

We then tested the therapeutic potential of mAb CC4 against human colorectal tumor in an *in vivo* system. Xenografts were established by injecting colorectal cancer LS174T cells to nude mice. These mice started to receive treatment of mAb CC4 or normal mIgG, administrated intraperitoneally, when the tumor reached a diameter of 3 to 5 mm. After observing both experimental and control groups for more than a month, we found that mice injected with mAb CC4 developed tumor as small as less than 0.2 cm^3^, however, tumors had grown to a very large size in mice treated with mIgG (Figure 2D). These data indicated that just naked mAb CC4, without being radiolabeled or anticancer agent-conjugated, was able to remarkably suppress human tumor growth, suggesting its application value in cancer therapy.

### ADCC activity of mAb CC4

We next explored the mechanisms underlying the anti-tumor activity of mAb CC4. First, we examined mAb CC4′s activity of inducing antibody-dependent cell cytotoxicity (ADCC), since it was reported that anti-CEA mAb hMN-14 (labetuzumab) inhibited colonic carcinoma growth mainly by raising ADCC reaction [19]. Isolated murine spleen cells were used as effector cells, while LS174T cells served as target cells. The ADCC activity of mAb CC4 was measured by LDH release based on a Homogeneous Membrane Integrity Assay. As shown in Figure 2E, mAb CC4 raised a strong cytotoxicity against LS174T cells by activating murine immune cells, compared to normal mIgG, however, it had no such effect on CC4-negative melanoma A375 cells. Therefore, the marked inhibition of xenograft tumor growth in mice by mAb CC4 could be partially explained by strong ADCC reactions.

### mAb CC4 inhibits cell proliferation and blocks cell migration and aggregation

**Figure 3:**
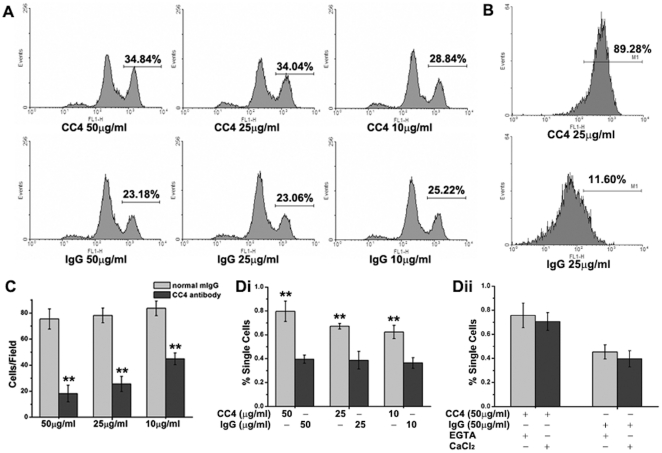
mAb CC4 suppressed proliferation, migration and aggregation of colorectal cancer cells. LS174T cells treated with indicated concentrations of mAb CC4 were subjected to proliferation assays using CFSE staining (A). The same assay was also performed using Lovo cells under the treatment of 25 µg/ml mAb CC4 (B). LS174T cells were used in migration assays using transwell system (C) and aggragation assays in the presence of indicated concentrations of mAb CC4 or normal mIgG (Di) with Ca^2+^ or EGTA (Dii). Statistically significant differences between mAb CC4 treated groups and control groups were indicated by double stars (**P<0.01).

We also examined the direct effects of mAb CC4 on colorectal cancer cells growth *in vitro*. 25 µg/ml of mAb CC4 is capable of significantly inhibiting the proliferation of CFSE-labeled colorectal cancer LS174T and Lovo cells (Figure 3A and Figure 3B). Further cell cycle analysis showed that mAb CC4 suppresses cell growth by abrogating S/G2 transition and preventing more than 15% cells from entering G2 phase (Figure S1). Growth of two other colorectal tumor cell lines, Colo-205 and SW1116, was also found suppressed by mAb CC4 (data not shown). Nevertheless, mAb CC4 does not markedly induce cancer cell apoptosis in TUNEL assays (Figure S2).

CEACAM5 mediates cell-cell contact by homophilic and heterophilic interaction and is also involved in cell adhesion and migration [3], [20]. Thus, we used chamber assay to find that mAb CC4 inhibited LS174T cells, Lovo cells and Colo-205 cells migration in a dose-dependent manner (Figure 3C and Figure S3). Furthermore, in a standard cell aggregation assay, around eighty percent of LS174T cells could not aggregate in the presence of 50 µg/ml mAb CC4, whereas only 40% of cells maintained single when treated with murine IgG (Figure 3Di). Adding chelating reagent EGTA or CaCl_2_ could not affect the function of mAb CC4 in blocking cell aggregation (Figure 3Dii), consistent with that CEACAM5 belongs to immunoglobulin superfamily and mediates Ca^2+^-independent cell-cell adhesion. Since colorectal cancer cells grow in aggregates morphologically in tissue culture, it is possible that mAb CC4 mainly influences tumor cell growth by disturbing cell-cell contact and interfering with signaling transduction. Similar results were also obtained in SW1116, Lovo and Colo-205 cells (data not shown).

### Epitope mapping of mAb CC4

**Figure 4:**
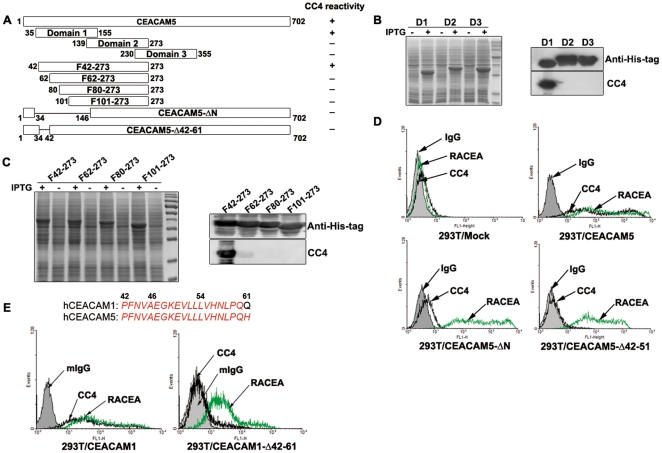
Epitope mapping of mAb CC4. Recombinant CEACAM5 fractions and full-length deletion mutants were illustrated in (A). These recombinant fractions were expressed in E. Coli and whole cell lysates were separated by SDS-PAGE and analyzed by immunoblot with anti-His-tag (positive control) and mAb CC4 (B and C). Plasmid expressing full-length CEACAM5 wild-type or mutants were transfected into 293T cells and these cells were then subjected to flow cytometry assays using mAb CC4 or rabbit polyclonal anti-CEA (RACEA) or mIgG (D). Amino acid sequences of region aa42–61 of CEACAM1 and CEACAM5 were shown; and 293T cells were transfected with plasmids expressing CEACAM1 wt or mutant and analyzed by flow cytometry with indicated antibodies (E).

Functions of mAb CC4 in directly affecting cell proliferation and migration led us to speculate that it might recognize a distinct epitope harboring key amino acids for the molecular functions. CEACAM5 consists of seven extracellular Ig domains, of which the N-terminal IgV-like domain is the most important and functional part in mediating CEACAM5 homophilic and heterophilic adhesions [7]. To test whether mAb CC4 could bind to the N-domain of CEACAM5 and potentially block its function, epitope mapping was performed using various extracellular fractions and mutants of the protein (Figure 4A).

Three extracellular Ig domains proximal to the N-terminus, also known as N, A1, and B1 domain, designated here as Domain1 (D1, aa35–155), Domain2 (D2, aa139–273) and Domain3 (D3, 230–355) respectively, were expressed; and only D1 was recognized by mAb CC4, indicating the epitope is located in the region of aa35–138 (Figure 4B). This region was further fractionized to generate four fragments, F42–273, F62–273, F80–273, and F101–273. Western blot results showed that mAb CC4 only bound to F42–273, suggesting the epitope is harbored in a narrowed region, aa42–61 (Figure 4C). These observations were then confirmed using full-length CEACAM5 mutants lacking N-domain or aa42–61. Flow cytometry assays showed that mAb CC4 could bind to 293T cells transfected with CEACAM5 but not cells expressing either CEACAM5-ΔN or CEACAM5-Δ42-61 (Figure 4D). Rabbit polyclonal anti-CEA antibody (RACEA) served as a positive control for the transfection. Therefore, these results revealed that the epitope of mAb CC4 is located in the functional N-domain of CEACAM5, more specifically the region of aa42–61.

Moreover, since the amino acid sequence of the epitope recognized by mAb CC4 is completely conserved between CEACAM5 and CEACAM1, mAb CC4 also binds to the 293T cells expressing wild-type CEACAM1 but not the mutant lacking aa42–61 (Figure 4E). The recognition of mAb CC4 to the same epitope in the N-domain of both CEACAM5 and CEACAM1 implicated that mAb CC4 may not only block CEACAM5-CEACAM5 homophilic interactions but also obstruct CEACAM1-CEACAM1 homophilic and CEACAM1-CEACAM5 heterophilic associations.

### Enhancement of NK killings against HLA-I^low^ colorectal cancer cells by mAb CC4

Above findings led to the possibility that mAb CC4 may interfere with the cell-cell contact between CEACAM1 expressed on immune cells and CEACAM5 expressed on colorectal cancer cells. In fact, both CEACAM1 and CEACAM5 expressed on melanoma or 721.221 cells suppress NK cell-mediated cytotoxicity against tumor cells by interacting with and activating NK CEACAM1, which is a MHC I-independent inhibitory receptor [17], [18]. However, this mechanism has not been tested in CEACAM5-overexpressing colonic adenocarcinoma.

**Figure 5:**
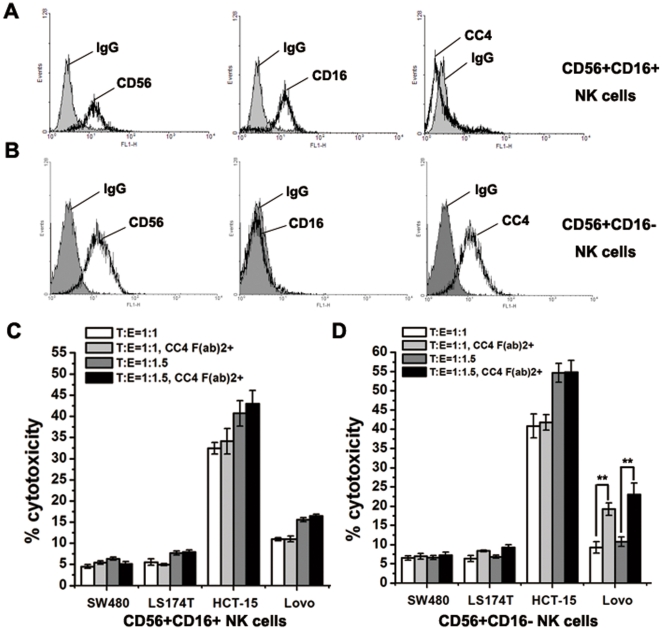
mAb CC4 enhanced the killing of HLA-I^low^ colorectal cancer cells by CD16- NK cells. CD56+CD16+ (A) and CD56+CD16- NK cells (B) isolated from human PBMCs were analyzed by flow cytometry. NK cytotoxicity assays were performed against various colorectal cancer cell lines using either CD56+CD16+ (C) or CD56+CD16- (D) NK cells as effector cells. Significant differences between mAb CC4 treatments and controls were indicated by double stars (**P<0.01).

We first screened ten different colorectal cancer cell lines for expression patterns of CEACAM5 and HLA-I by flow cytometry analysis (Table S3), and chose four cell lines with different expression patterns for further studies, SW480 (CC4-, HLA-I^high^), LS174T (CC4+, HLA-I^high^), HCT-15 (CC4-, HLA-I^low^) and Lovo (CC4+, HLA-I^low^). Both CD56+CD16+ and CD56+CD16- Human NK cells were isolated from human PBMCs (peripheral blood mononuclear cells) using magnetic cell sorting system. Cell types were verified by staining with fluorescent antibodies and it was found that only CD56+CD16- NK cells but not CD56+CD16+ cells express CEACAM1 (Figure 5A and B). These NK cells were subjected to cytotoxicity assays against various colorectal cancer cells. In order to avoid any possible ADCC activity, mAb CC4 was digested with pepsin and the resulted CC4 F(ab)_2_ fragment was used in the experiments.

Owing to the high expression levels of HLA-I molecules both SW480 and LS174T were not killed by NK cells, while HCT-15 cells lacking HLA-I were killed efficiently and adding CC4 F(ab)_2_ had no effect on cytotoxicity since HCT-15 does not express CEACAM5 (Figure 5C and D). However, HLA-I-deficient but CEACAM5-positive Lovo cells could not be readily killed by CEACAM1-expressing CD56+CD16- NK cells; and the treatment of CC4 F(ab)_2_ significantly enhanced the cytotoxicity against Lovo cells by CD16- NK cells but not CD16+ NK cells. These results indicated that mAb CC4 could specifically promote the killing against CEACAM5-expressing cells by CEACAM1-expressing NK cells.

### Disruption of CEACAM1-mediated inhibition of NK activity by mAb CC4

**Figure 6:**
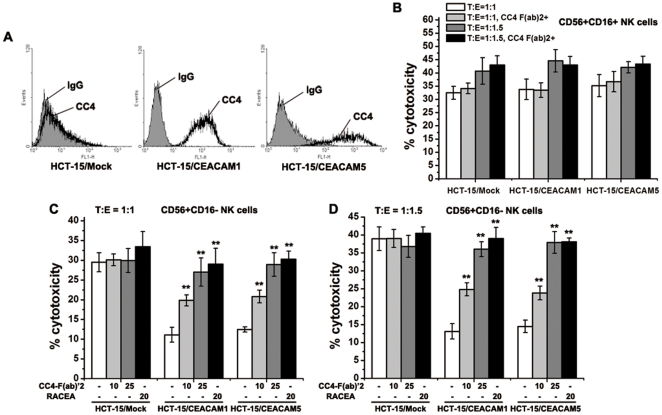
mAb CC4 blocked the suppression of NK killings induced by over-expression of CEACAM5 in colorectal cancer cells. Forced expressions of CEACAM1 and CEACAM5 in HCT-15 cells were confirmed by flow cytometry (A). Stable transfectants were then subjected to NK cytotoxicity assays using CD56+CD16+ NK cells (B) and CD56+CD16- NK cells under different ratios of numbers of target cells to that of effector cells (C and D). Significant differences between mAb CC4 treated or RACEA-treated groups and the corresponding control groups were indicated by double stars (**P<0.01).

To further verify that mAb CC4-enhanced NK killings is based on the disruption of CEACAM1-CEACAM5 interaction between NK and tumor cells. We transfected CEACAM1- and CEACAM5-expressing plasmids into colorectal cancer HCT-15 cells, respectively, and then generated stable cell lines, designated as HCT-15/CEACAM1 and HCT-15/CEACAM5. Forced expressions of these molecules in the transfectants were confirmed by flow cytometry analysis (Figure 6A).

Increasing the expressions of either CEACAM1 or CEACAM5, the ligand for NK CEACAM1, did not affect the killings against cancer cells by CD56+CD16+ NK cells, since these cells do not express CEACAM1 (Figure 6B). However, the cytotoxicity induced by CEACAM1-expressing CD56+CD16- NK cells was significantly compromised by ectopic expression of CEACAM1 or CEACAM5 in HCT-15 cells (Figure 6C and D). Thus, the tumor immune evasion mechanism that up-regulation of CEACAM1 or CEACAM5 activates inhibitory CEACAM1 signaling in NK cells and then circumvents NK cytotoxicity also exists in colorectal cancer cells. More importantly, treatment of CC4 F(ab)_2_ remarkably blocked the repression of NK killing activity caused by either CEACAM1 or CEACAM5 ectopic expression, suggesting that the effect of mAb CC4 in enhancing the immune reaction against tumor cells is attributed to its function of specifically abrogating the initiation of CEACAM1 signaling in NK cells. Notably, mAb CC4 enhances NK cytotoxicity in a dose-dependent manner and has a comparable activity as the neutralizing polyclonal antibodies RACEA.

## Discussion

With the advantages of targeting tumor cells specifically, monoclonal antibodies against tumor-associated antigens provide great opportunities for biological therapy of cancers. Following the discovery of Carcinoembryonic antigen (CEA or CEACAM5) as a colorectal cancer-associated antigen, anti-CEACAM5 mAbs have been found effective in diagnosis and therapy of multiple types of cancers. A humanized anti-CEACAM5 mAb, labetuzumab, and human monoclonal antibodies (HmAbs) were found able to suppress tumor growth and metastasis [21], [22], while other mAbs labeled with radioactive isotopes were applied for tumor imaging [23], [24]. However, nearly no single anti-CEACAM5 mAb has been reported to have both tumor-targeting and tumor-suppressing activities. In this study, we reported a novel anti-CEACAM5 mAb CC4, which has striking capabilities of both targeting and accumulating in tumor tissues *in vivo* and remarkable functions of inhibiting tumor growth in animal models. These capacities of mAb CC4 make it a promising diagnostic tool as well as a potential therapeutic for the treatment of cancers, particularly colorectal cancer.

CEACAM5 mainly mediates cell adhesion and enhances cell migration through homophilic binding or heterophilic interaction with other CEACAMs. These functions are disturbed by mAb CC4 according to its capabilities of inhibiting cell aggregation and transmigration. Interestingly, it was reported that intercellular adhesion of CEACAM5 requires N-terminal IgV-like domain, more precisely three subdomains: GYSWYK (aa64–69), NRQII (aa76–80) and QNDTG (aa114–118) [7], while mAb CC4 recognizes an epitope (aa42–61) very close to these amino acids. Thus, the binding of mAb CC4 to CEACAM5 may interfere with its interactions with itself or other CEACAMs structurally or topologically. It is worth noting that 10 µg/ml of mAb CC4 is sufficient to block cell aggregation whereas peptides targeting these subdomains need a much higher minimal concentration (1 mg/ml) to block the adhesion [25]. High efficiency of mAb CC4 might be attributed to its high stability and affinity to the interface of CEACAM5 homophilic interactions.

Moreover, mAb CC4 also shows some interesting functions of directly affecting tumor cell growth, such as suppressing colorectal cancer cell proliferation. These properties were not observed in the studies of other anti-CEACAM5 therapeutic mAbs, such as labetuzumab [19]. Though there is a strong correlation between CEACAM5 expression levels and tumor growth, little is known about its role in promoting proliferation. It will be of interest to study why mAb CC4 could influence cell proliferation through binding to CEACAM5.

Tumor cells adopt various mechanisms to escape from cancer immunosurveillance mediated by lymphocytes of both the adaptive and innate immune compartments [26]. One of the widely found strategies in tumor is the depletion or mutations of MHC class I molecules in transformed cells in order to avoid presenting tumor antigens and bypass the attack of adaptive immune systems [27]. However, NK cytotoxicity could be activated against these “non-self” cells lacking specific class I MHC [28]. Identification of CEACAM1 as a MHC-I-independent NK inhibitory receptors provided new insights toward understanding tumor immune evasion mechanisms [17], since the binding partners of CEACAM1—CEACAM1 itself and CEACAM5—are widely present in various types of cancers. It was first identified in MHC-I-negative melanoma cells (1106mel) that expression of CEACAM1 blocked the killing by CD16-CEACAM1+ NK cells [17]. In present study, we found that some HLA-I-deficient colorectal cancer cells (Lovo) present high levels of CEACAM5 and were resistant to the killings by CD16-negative NK cells. Importantly, overexpression of either CEACAM1 or CEACAM5 in CEACAM5-HLA-I^low^ colonic tumor cells could prevent NK cytotoxicity against these cells. These data indicated that similar mechanisms by which cancer cells lacking class I MHC molecules express CEACAM1 ligands to suppress the killing activity of NK cells are also adopted by colorectal tumor cells and might be common in all kinds of cancers.

The finding that CEACAM5 protects tumor cells from NK killing is rather significant in colorectal cancers since a very high percentage of colonic adenocarcinomas display strong expressions of CEACAM5 [29]. Therefore, it offers a target for blocking tumor immune escape. We showed here that mAb CC4 could markedly enhance NK cytotoxicity against colorectal cancer cells by disrupting the inhibitory interaction of tumor CEACAM5 or CEACAM1 with NK CEACAM1. Monoclonal antibodies against CEACAMs may provide new routes for cancer immune therapy.

## Materials and Methods

### Ethics Statement

Human tumor tissues and normal tissues were collected under a protocol approved by the Institutional Review Board of the Institute of Biophysics, Chinese Academy of Sciences, that included the use of biopsy material for further biological studies. In accordance, the tissue donor gave a written informed consent that included the use of tumor material and normal tissue for cell banking as well as for the establishment of the tumor cell line and use of the cells for further studies. Maintenance of mice and experimental procedures were reviewed and approved by the Animal Welfare and Research Ethics Committee of the Institute of Biophysics, Chinese Academy of Sciences (Permit No.: SYXK2009-103). Mice were housed in a specific pathogen-free barrier facility. Surgeries were performed under sodium pentobarbital anesthesia, and all efforts were made to minimize suffering. All animals were euthanized after the experiments to allow the collection of tumor tissues.

### Cells, tissues and animals

All the cell lines were obtained from American Type Culture Collection (Rockville, MD). Both CD56+CD16+ and CD56+CD16- Human NK cells were sorted out from human blood-derived peripheral blood mononuclear cells (PBMCs) using magnetic cell sorting kits (Miltenyi Biotec, Germany). Briefly, PBMCs were isolated by density gradient centrifugation using Ficoll-Paque (GE Healthcare). The isolation of CD56+CD16+ NK cells was performed in a two-step procedure. First, non-NK cells such as T cells, B cells, dendritic cells, stem cells, monocytes, granulocytes, and erythroid cells were indirectly magnetically labeled with a cocktail of biotin-conjugated antibodies against lineage-specific antigens and a cocktail of MicroBeads, Upon subsequent magnetic separation of the cells over a MACS Column that was placed in a magnetic field, the magnetically labeled non-NK cells were retained within the column while the unlabeled NK cells ran through. In the second step, the pre-enriched NK cells were directly labeled with CD16 MicroBeads. Upon subsequent magnetic separation, the CD56+CD16+ NK cells were eluted after removing the column from the magnetic field. Similar methods were also used to isolate CD56+CD16- NK cells. Non-NK cells and CD56+CD16+ NK cells were depleted in the first step, and CD56+CD16- NK cells were then directly labeled with CD56 MicroBeads and magnetically separated in the second step. Both kinds of NK cells were cultured in alpha-MEM with 10% horse serum and 1 mM glutamate, 1 mM non-essential amino acid, 1 mM sodium pyruvate, 2×10^−5 ^M b-ME and 50 U/ml recombinant human Interleukin-2. Human tumor tissues were obtained from the tissue bank of the 301 Hospital in Beijing, and normal tissues are obtained from the Beijing Legal Medial Institute. 6–8 week-old female inbred BALB/c mice and nude mice were obtained from the Animal Center of the Chinese Academy of Medical Science, Beijing.

### Antibodies and constructs

The primary antibodies used were polyclonal rabbit-anti-human Carcinoembryonic antigen (DakoCytomation), anti-HLA-ABC (Abcam), isotype-matched IgG (Sigma). Mouse mAb CC4 was purified from ascites using protein A-Sepharose. Corresponding species-specific biotinylated, FITC-conjugated (Dianova, Hamburg, Germany) or HRP-conjugated (Pierce) secondary antibodies were used. For labeling antibodies, one mg of purified CC4 antibodies was labeled with Cyanine 5 using the Cy5 NHS ester (Amersham).

pcDNA3.1-CEACAM5, pcDNA3.1-CEACAM5ΔN and pcDNA3.1-CEACAM1 are generous gifts from Dr. Ofer Mandelboim (Hebrew University of Jerusalem). Different fragments of CEACAM5 extracellular domains were cloned from CEACAM5 cDNA into pET32a correspondingly to generate the constructs expressing recombinant CEACAM5 fractions fused with Trx-His6-S tag. Full-length mutants of CEACAM5 and CEACAM1 were generated by standard over-lapping PCR.

### Generation and screening of monoclonal antibody

1×10^7^ of human colorectal cancer cells, LS174T, were injected intraperitoneally with Freund complete adjuvant into 6-week-old BALB/c mice and boosted four times at two-week intervals, and then their spleens were taken for hybridoma preparation by fusion with mouse SP2/0-Ag14 myeloma cells as described [30]. ELISA was used to select antibodies binding to LS174T cells. Briefly, cells were grown in 96-well plates and fixed in pre-chilled methanol/acetone (1∶1). Plates were then blocked in 5% non-fat milk/PBS and incubated with hybdridoma culture supernatants, followed by washings and incubation with HRP-conjugated anti-mouse IgG (Sigma). Color reaction, developed by adding TMB (Sigma) as substrate, was measured by a BioRad ELISA reader at 450 nm.

### Flow cytometry

Detached cells were processed to obtain single-cell suspensions followed by staining with CC4 or anti-CEA antibodies on ice for 40 minutes. After 3 times of washing with PBS containing 0.3% bovine serum albumin, cells were incubated with corresponding fluorescein isothiocyanat (FITC) conjugated anti-mouse IgG (Sigma) on ice for 30 minutes, then washed and analyzed using a FACS Calibur flow cytometry system (Becton Dickinson, San Jose, CA).

### Immunoprecipitation and antigen identification

LS174T cells were collected and lysed in ice-cold RIPA buffer [Tris-HCl 50 mM pH 8.0, NaCl 150 mM, EDTA 1 mM, NP-40 1%, Glycerol 10%, PMSF 100 µg/ml]. After centrifugation the supernatant was pre-cleared with protein A-Sepharose (Sigma), and diluted cell lysate (1 mg/ml of total proteins) was then immunoprecipitated with either mAb CC4 or mIgG at 4°C for 2 h, followed by incubation with protein A-Sepharose for 1 h. After extensively washings, immunoprecipitates were analyzed by Western blot assay.

Antigen identification and proteomic analysis were performed as described by Gharahdaghi et al [31]. Briefly, protein captured by mAb CC4 was separated by 10% SDS-PAGE and visualized by silver staining. Protein bands smearing from 140 kD to 110 kD were excised manually and standard trypsin digestion and subsequent liquid chromatography-mass spectrometric (LC-MS) analysis of the protein digests were performed. The peptide sequences thus obtained were searched for in the NCBI and SwissProt Databanks using Mascot Deamon software (Matrix Science, London, UK).

### CFSE staining

In proliferation assay, LS174T cells were resuspended in PBS/0.1% BSA at a concentration of 1×10^6^ cells/ml and labeled with 10 µM CFSE at 37°C for 10 minutes. Cells were then seeded in 6 well plates (1.5×10^6^ cells/well), and 6 hours later different concentrations of mAb CC4 or mIgG were added. 18 hours later, cells were harvested and analyzed using a flow cytometer with 488 nm excitation and emission filters appropriate for fluorescein.

### Transmigration and aggregation blocking assay

Cell transmigration was assayed using transwells (8-µm pore size; Corning Costar, NY). Cells were resuspended in serum-free medium containing 1% BSA and then added to the upper chamber (10000/well) in the presence of either mAb CC4 or mIgG. Lower chambers contained 20% fetal calf serum (FCS). After incubation at 37°C overnight, cells remained at the upper surface of the membrane were removed using a swab, while the cells that migrated to the lower membrane surface were fixed with ethanol and stained with Giemsa solution. Cells migrating through the filter were counted and the number of migrating cells per optic field (×20) was plotted.

Cell aggregation assay is based on a previously described method [32]. Subconfluent cell layers were detached by incubation in Hank's Buffered Salt Solution (HBSS) containing 1 mM EDTA. Cells were washed, resuspended (5×10^5^ cells/ml) in HBSS/BSA by three passages through an 18-gauge needle and then seeded in a 24-well plate previously coated with 2% BSA in HBSS and allowed to aggregate for 3 hours in the presence of mAb CC4 or mIgG at 37°C on a rotating shaker (80 rpm). The reaction was stopped by the addition of 0.5 ml of 25% glutaraldehyde per well. Aggregation was quantified by counting representative aliquots from each sample. The blockage of aggregation was quantified by the following formula: % Single cells = N_S_/N_0_ × 100, where Ns is the total number of single cells, and N_0_ is the total number of cells. At least 600 cells were counted from each sample.

### ADCC and NK cytotoxicity assay

The method of LDH release was used in both cytotoxicity analyses. Briefly, for ADCC assay 5000 cells of LS174T or A375 were grown in 96-well plate as the target cells. 5×10^5^ mouse spleen cells, isolated from BALB/c mice, were added (T∶E = 1∶100) with different concentrations of mAb CC4 or mIgG. For NK cytotoxicity assay, 10, 000 cancer cells were used as target cells and 10, 000 or 15, 000 NK cells (T∶E  = 1∶1 or 1∶1.5) were added in the presence of CC4 F(ab)_2_ or anti-CEA polyclonal antibody RACEA. Four hours later, enzymatic activity of LDH released from target cells were measured by CytoTox-ONE Homogeneous Membrane Integrity Assay kit (Promega). Cytotoxicity was quantified by the following formula: % cytotoxicity = (Exp – T spon – E spon) / (T max – T spon), in which T spon and E spon are the spontaneous LDH release of target and effector cells respectively, and T max is the maximum of LDH release of target cells, obtained by lysing them before testing.

### In vivo optical imaging of tumor-bearing mice

Subcutaneous (s.c.) injection of 2×10^7^ A549 cells suspended in 200 µl of PBS into the right flank of female athymic nude mice resulted in formation of tumors after 4–6 weeks. The mice bearing s.c tumors at diameter of 6–8 mm were used for imaging experiments. They received intravenous (i.v.) injection of Cy5-labeled CC4 antibody at 20 µg/200 µl for each mouse or the isotype Cy5-IgG negative control at a similar concentration. Fluorescence reflectance imaging was performed using a Hamamatsu optical imaging system as described previously [33]. For quantifying tumor contrast, the mean fluorescence intensities of the tumor area (T) and that of the distant skin area (S) were calculated; dividing T by S produced the ratio between tumor tissues and background level.

### Human colorectal cancer xenografts

Xenografts of human colorectal cells were produced by injecting LS174T cells (1×10^7^ resuspended in PBS) subcutaneously into the back of 6-week-old BALB/c nude mice. When tumors reached a diameter of 3 to 5 mm, the mice were grouped (7 mice per group) and administrated intraperitoneally with purified mAb CC4 at a dose of 5 mg/kg or PBS, twice per week for 21 to 28 days. Tumor size was measured twice per week and tumor volume was determined according to the equation: tumor volume = width^2^×length× (π/6). The 2-tailed Student t-test was used for statistical analysis.

### Statistical analysis

All values are representative of experiments performed in triplicate. The graphical results are expressed as the mean ± SD. Paired t test methods were used to compare differences between groups in various experiments. The criterion for statistical significance is defined as p<0.01 (double stars) or 0.01<p<0.05 (single star).

Additional experimental methods can be found in Supplemental Materials and Methods (Text S1).

## Supporting Information

Figure S1 **mAb CC4 repressed cell cycle transition of LS174T cells.** LS174T cells were serum-starved for 48 hours to synchronize cell cycle and then treated with indicated concentrations of mAb CC4 or normal murine IgG for 12 hours and subjected to cell cycle analysis. Percentages of cells remained in G1, S and G2 phases were calculated by the software of Cylchred and presented in (A)–(D).
(TIF)

Figure S2 **mAb slightly induced apoptosis of LS174T cells.** Cells were treated with 50 µg/ml mAb CC4 or mIgG for 24 hours and then subjected to TUNEL assay to label the DNA strand breaks, the indicator for apoptosis. The nuclei were stained by DAPI. Typical optic fields (×10) of treated cells were presented in (A) and apoptotic cells were seen in fluorescent green. The mean values of numbers of apoptotic cells per 100 cells were presented in bar graph (B). At least ten fields were calculated and included in statistic analysis.
(TIF)

Figure S3 **mAb CC4 inhibited colorectal cancer cell migration.** Transmigration assays were applied using Lovo cells (A) and Colo-205 cells (B) in the presence of indicated concentrations of mAb CC4 or normal murine IgG. The number of cells migrating through the filter was countered and plotted as the number of migrating cells per optic field (×20).
(TIF)

Table S1 **Flow cytometry and immunofluorescent analysis of mAb CC4 immunoreactivity to human cancer cell lines.**
(DOCX)

Table S2 **Immunohistochemical analysis of the specificity of mAb CC4 for normal and tumor human tissues.**
(DOCX)

Table S3 **Fluorescent intensity of mAb CC4 and anti-HLA-ABC immunostaining against human colorectal cancer cell lines.**
(DOCX)

Text S1 **Supplemental** **Materials and Methods**
(DOCX)
