# Supplementary figures and images for: A Novel Anti-CEACAM5 Monoclonal Antibody, CC4, Suppresses Colorectal Tumor Growth and Enhances NK Cells-Mediated Tumor Immunity
Source: PLoS One. 2011 Jun 22;6(6):e21146. doi: 10.1371/journal.pone.0021146 (PMC3120848; doi:10.1371/journal.pone.0021146)

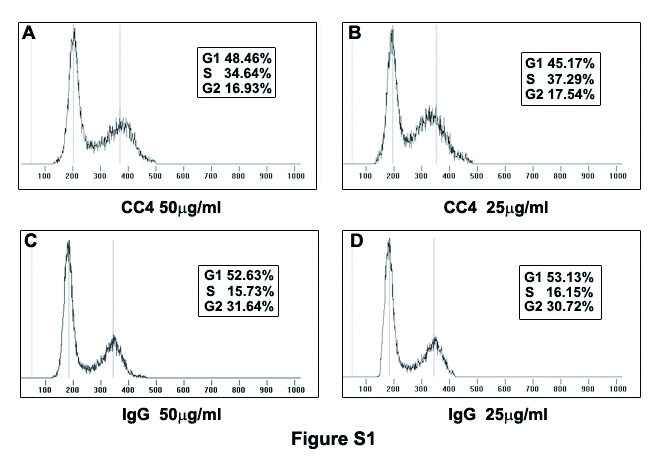

Supplement: Figure S1 — mAb CC4 repressed cell cycle transition of LS174T cells. LS174T cells were serum-starved for 48 hours to synchronize cell cycle and then treated with indicated concentrations of mAb CC4 or normal murine IgG for 12 hours and subjected to cell cycle analysis. Percentages of cells remained in G1, S and G2 phases were calculated by the software of Cylchred and presented in (A)–(D). (TIF) [file pone.0021146.s001.tif]

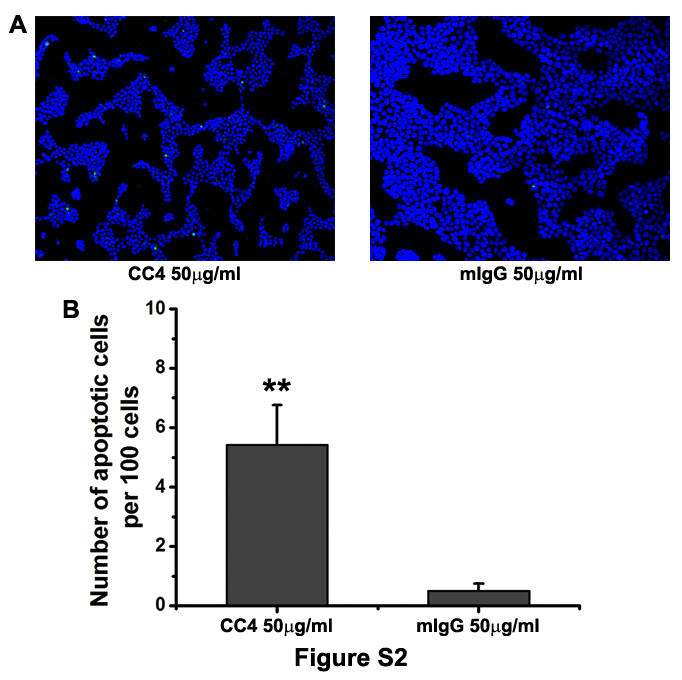

Supplement: Figure S2 — mAb slightly induced apoptosis of LS174T cells. Cells were treated with 50 µg/ml mAb CC4 or mIgG for 24 hours and then subjected to TUNEL assay to label the DNA strand breaks, the indicator for apoptosis. The nuclei were stained by DAPI. Typical optic fields (×10) of treated cells were presented in (A) and apoptotic cells were seen in fluorescent green. The mean values of numbers of apoptotic cells per 100 cells were presented in bar graph (B). At least ten fields were calculated and included in statistic analysis. (TIF) [file pone.0021146.s002.tif]

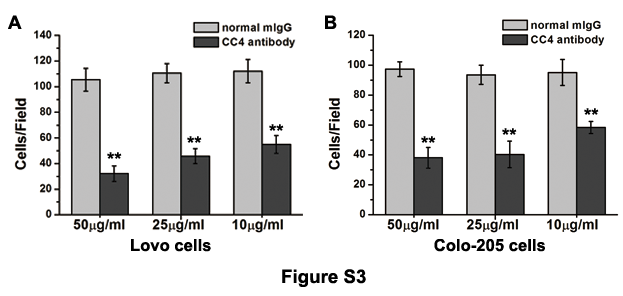

Supplement: Figure S3 — mAb CC4 inhibited colorectal cancer cell migration. Transmigration assays were applied using Lovo cells (A) and Colo-205 cells (B) in the presence of indicated concentrations of mAb CC4 or normal murine IgG. The number of cells migrating through the filter was countered and plotted as the number of migrating cells per optic field (×20). (TIF) [file pone.0021146.s003.tif]
