# Supplementary material for: A Novel Anti-CEACAM5 Monoclonal Antibody, CC4, Suppresses Colorectal Tumor Growth and Enhances NK Cells-Mediated Tumor Immunity
Source: PLoS One. 2011 Jun 22;6(6):e21146. doi: 10.1371/journal.pone.0021146 (PMC3120848; doi:10.1371/journal.pone.0021146)
Supplement: Table S1 — Flow cytometry and immunofluorescent analysis of mAb CC4 immunoreactivity to human cancer cell lines. (DOCX) [file pone.0021146.s004.docx]

Table S1 Flow cytometry and immunofluorescent analysis of mAb CC4 immunoreactivity to human cancer cell lines.

| cell line | mAb CC4 |
| --- | --- |
| Bladder cancer cell T24 | - |
| Bladder cancer cell 5637 | - |
| Colorectal cancer cell HT-29 | + |
| Colorectal cancer cell LS 174T | + |
| Colorectal cancer cell SW1116 | + |
| Colorectal cancer cell SW948 | + |
| Colorectal cancer cell SW480 | + |
| Colorectal cancer cell SW620 | + |
| Colorectal cancer cell Colo-205 | + |
| Colorectal cancer cell Colo-320 | + |
| Colorectal cancer cell Lovo | + |
| Hepatocarcinoma ALEX | - |
| Hepatocarcinoma HepG2 | + |
| Choriocarcinoma Bewo | - |
| Breast cancer cell MCF-7 | - |
| Breast cancer cell ZR-75-1 | - |
| Non small cell lung cancer cell H460 | - |
| Non small cell lung cancer cell H1299 | - |
| Non small cell lung cancer cell SK-LU-1 | + |
| Non small cell lung cancer cell PG | + |
| Non small cell lung cancer cell 95D | - |
| Melanoma A375 | - |
| Pancreatic cancer Capan-2 | + |
| Pancreatic cancer SW1990 | + |
| duodenal cancer cell HuTu 80 | - |

+ indicates positive for mAb AA98; -, negative.
