# Supplementary material for: A Novel Anti-CEACAM5 Monoclonal Antibody, CC4, Suppresses Colorectal Tumor Growth and Enhances NK Cells-Mediated Tumor Immunity
Source: PLoS One. 2011 Jun 22;6(6):e21146. doi: 10.1371/journal.pone.0021146 (PMC3120848; doi:10.1371/journal.pone.0021146)
Supplement: Table S2 — Immunohistochemical analysis of the specificity of mAb CC4 for normal and tumor human tissues. (DOCX) [file pone.0021146.s005.docx]

Table S2 Immunohistochemical analysis of the speciﬁcity of mAb CC4 for normal and tumor human tissues.

| Tissues | mAb CC4 +/cases |
| --- | --- |
| Normal tissues | |
| Stomach | 1/3 |
| Colon | 2/9 |
| Liver | 2/2 |
| Uterus | 3/8 |
| Brain | 0/2 |
| Heart | 0/3 |
| Spleen | 2/2 |
| Pancreas | 0/2 |
| Kidney | 2/3 |
| Thyroid gland | 0/1 |
| Bladder | 0/1 |
| Prostate | 1/2 |
| Testis | 0/1 |
| Mammary gland | 0/1 |
| Lung | 0/1 |
| Tumors | |
| Gastric carcinoma | 9/9 |
| Colorectal carcinoma | 15/15 |
| Hepatocarcinoma | 8/11 |
| Endometrial cancer | 7/9 |
| Lung cancer | 8/9 |
| Oesophageal cancer | 8/8 |
| Ovary cancer | 4/4 |
| Breast cancer | 5/7 |
| Cerebral cancer | 0/8 |
| Renal carcinoma | 3/8 |
| Prostatic carcinoma | 2/8 |
