# Supplementary material for: A Novel Anti-CEACAM5 Monoclonal Antibody, CC4, Suppresses Colorectal Tumor Growth and Enhances NK Cells-Mediated Tumor Immunity
Source: PLoS One. 2011 Jun 22;6(6):e21146. doi: 10.1371/journal.pone.0021146 (PMC3120848; doi:10.1371/journal.pone.0021146)
Supplement: Table S3 — Fluorescent intensity of mAb CC4 and anti-HLA-ABC immunostaining against human colorectal cancer cell lines. (DOCX) [file pone.0021146.s006.docx]

Table S3 Fluorescent intensity of mAb CC4 and anti-HLA-ABC immunostaining against human colorectal cancer cell lines.

|  | background | CC4 | HLA-1 |
| --- | --- | --- | --- |
| LS174T | 3.68 | 92.08 | 816.27 |
| SW948 | 3.33 | 15.72 | 947.91 |
| SW1116 | 3.93 | 42.43 | 850.82 |
| SW620 | 3.21 | 14.9 | 170.78 |
| SW480 | 3.12 | 6.41 | 340.33 |
| HCT-15 | 3.85 | 4.59 | 2.79 |
| Lovo | 3.43 | 97.21 | 3.58 |
| Colo-205 | 3.2 | 48.6 | 322.69 |
| Colo-320 | 2.46 | 13.04 | 78.39 |
| HT-29 | 3.16 | 6.76 | 744.98 |
