# Supplementary material for: A Novel Anti-CEACAM5 Monoclonal Antibody, CC4, Suppresses Colorectal Tumor Growth and Enhances NK Cells-Mediated Tumor Immunity
Source: PLoS One. 2011 Jun 22;6(6):e21146. doi: 10.1371/journal.pone.0021146 (PMC3120848; doi:10.1371/journal.pone.0021146)
Supplement: Text S1 — Supplemental Materials and Methods (DOCX) [file pone.0021146.s007.docx]

**Supplemental Materials and Methods**

***Western blot***

Cells were lysed in RIPA buffer [Tris-HCl 50mM pH 8.0, NaCl 150mM, EDTA 1mM, NP-40 1%, Glycerol 10%, PMSF 100µg/ml]; and soluble fractions were subjected to SDS-PAGE and then electrophoretically transferred to nitrocellulose membrane (Invitrogen). After blocking with 5% non-fat milk/PBST, membranes were incubated with primary antibodies, and then reacted with corresponding HRP-conjugated secondary antibody. The specific immunoreactive proteins were visualized with enhanced chemiluminescence reagent (Pierce, Rockford, IL).

***Immunohistochemistry and Immunofluoresence***

For immunohistochemistry, tissues specimens were cryosectioned, ﬁxed in acetone at -20°C for 10 minutes, and then preincubated with 0.3% H_2_O_2_ in methanol for 30 minutes to quench endogeneous peroxidases. Sequentially, the sections were blocked with 5% horse serum in PBS at 37^o^C for 30 minutes, and then incubated with primary antibodies in PBS overnight at 4^o^C. Appropriate biotin-conjugated secondary antibodies were applied followed by HRP-conjugated streptavidin. After every incubation step the sections were washed with PBS for 3 times. Freshly prepared 3, 3’-Diaminobenzidine (DAB) was added to the sections as substrate. The sections were finally counterstained with hematoxylin.

For immunifluoresence, cells were plated on coverslips and fixed with acetone: methanol (1:1) for 1 minute. After washing with PBS, cells were blocked with 5% normal goat serum for 30 minutes at 37°C, and then incubated with mAb CC4 overnight at 4°C, followed by incubation with FITC conjugated anti-mouse IgG for 30 minutes at 37°C. Finally, the coverslips were examined with a confocal laser scanning microscope (Olympus, Tokyo, Japan).

***Cell cycle assay***

For cell cycle assay, cells were serum-starved for 48 hours and then cultured in complete growth medium with mAb CC4 or mIgG for 12 hours, followed by washings in PBS and fixation in 70% ethanol at 4^o^C overnight. After centrifugation and washing with PBS, the cells were incubated with PI staining solution (0.1%Triton X-100, 200μg/ml DNase-free RNase A, 20μg/ml PI in PBS) for 30mins in the dark at room temperature and then analysed with a FacsCalibur.

***TUNEL assay***

For determining apoptotic rate of cancer cells induced by mAb CC4, TUNEL assay was carried out using ApopTag Fluorescein In Situ Apoptosis Detection Kit (Chemicon). Briefly, LS174T cells were plated on coverslips, treated with 50μg/ml mAb CC4 or normal murine IgG and then fixed with 4% para-formaldehyde at room temperature for 1 hour. After washings with PBS, cells were incubated in blocking buffer (3% H_2_O_2_ in methanol) for 10 minutes at room temperature and then incubated in equilibration buffer (0.1% Triton X-100 in 0.1% Sodium Citrate) for 30 minutes on ice. Sequentially, TdT enzyme reaction buffer (equilibration buffer with FITC-12-dUTP and TdT enzyme) was added. After incubation at 37^o^C for 60 minutes in the dark, cells were washed with PBS for three times and further stained with DAPI. The coverslips were examined with a confocal laser scanning microscope (Olympus, Tokyo, Japan) and the apoptotic cells labeled with FITC (green) were counted.
